# Reporting Data on Auditory Brainstem Responses (ABR) in Rats: Recommendations Based on Review of Experimental Protocols and Literature

**DOI:** 10.3390/brainsci11121596

**Published:** 2021-11-30

**Authors:** Ewa Domarecka, Mahmut Tayyar Kalcioglu, Ahmet Mutlu, Abdulkadir Özgür, Jasper Smit, Heidi Olze, Agnieszka J. Szczepek

**Affiliations:** 1 Department of Otorhinolaryngology, Head and Neck Surgery, Charité-Universitätsmedizin Berlin, Corporate Member of Freie Universität Berlin, Humboldt-Universität zu Berlin, 10117 Berlin, Germany; heidi.olze@charite.de; 2 Department of Otorhinolaryngology, Head and Neck Surgery, Faculty of Medicine, Istanbul Medeniyet University, 34720 Istanbul, Turkey; mtkalcioglu@hotmail.com (M.T.K.); ahmutlu1988@gmail.com (A.M.); 3 Otorhinolaryngology Clinic, Goztepe Prof. Dr. Suleyman Yalcin City Hospital, Kadikoy, 34722 Istanbul, Turkey; 4 Department of Otorhinolaryngology, İstanbul Yeni Yuzyil University Gaziosmanpaşa Hospital, 34245 Istanbul, Turkey; akozgur53@gmail.com; 5 Zuyderland Medical Center, Department of Otorhinolaryngology, Head and Neck Surgery, 6419 PC Heerlen, The Netherlands; ja.smit@zuyderland.nl; 6 Faculty of Medicine and Health Sciences, University of Zielona Góra, 65-046 Zielona Góra, Poland

**Keywords:** auditory brainstem responses, experimental conditions, rats, gender, strain

## Abstract

Research in hearing science is accelerating, and a wealth of data concerning auditory brainstem responses (ABR) in various animal models is published in peer-reviewed journals every year. Recently, we reviewed studies using ABR measurements in tinnitus rat models. We found significant discrepancies in the outcomes of these studies, some due to different research approaches and others due to different methodologies. Thus, the present work aimed to collect comprehensive information on all factors influencing ABR recordings in rats and compile recommendations on ABR data reporting. A questionnaire with queries about animal husbandry, transfer, handling, and the exact test conditions before, during, and after ABR recordings was sent to 125 researchers who published the relevant studies between 2015 and 2021. Eighteen researchers provided detailed answers on factors related to ABR measurements. Based on the analysis of the returned questionnaires, we identified three domains reflecting animal-, equipment-, and experiment-dependent factors that might influence the ABR outcome, thus requiring reporting in published research. The analysis of survey results led to the compilation of recommendations for reporting ABR outcomes supported by a literature review. Following these recommendations should facilitate comparative and meta-analyses of ABR results provided by various research groups.

## 1. Introduction

Hearing loss is the most common human sensory impairment, comprising various forms of hearing loss such as noise-induced hearing loss and age-related hearing loss [1,2]. Hearing loss enhances the risk of developing tinnitus and hyperacusis [3] and is associated with increased cognitive decline and dementia [4,5,6]. Thus, developing effective treatments to prevent and treat hearing loss has an urgent need and requires a complete understanding of the pathophysiology of the auditory system. The same holds for tinnitus and hyperacusis [7]. The knowledge of mechanisms involved in hearing impairment advanced considerably in the past decades of research, which is primarily based on animal models [8].

Progress in auditory research involves the development of various audiological methods. One of such methods involves electroencephalography (EEG) recordings of electrical potentials generated by auditory neurons in response to acoustic stimuli (auditory evoked potentials, AEP). A particular form of AEP recordings is auditory brainstem response (ABR) [8]. With ABR, hearing thresholds can be objectively assessed and measure changes in neural integrity in subcortical auditory structures. Therefore, ABR can be used to screen hearing thresholds and identify retrocochlear lesions, both in clinical practice and in animal research.

Basic research studies involving ABR use various animal models, but rats are used most frequently [9] based on similarities between humans and animal cochlear anatomy and physiology [10] as well as easy handling and low costs. In rodents, wave I is generated from the auditory nerve (distal portion). Waves II-V are believed to be associated with the cochlear nucleus, superior olivary complex, lateral lemniscus, and inferior colliculus, respectively [11]. Some studies suggested the involvement of the inferior colliculus in wave IV generation and the medial geniculate body of the thalamus in wave V generation [12,13].

Despite the frequent use of ABR in animal models, no universal guidelines are available, and not all factors that influence ABR are reported in published research. These factors include animal strain, gender, age, and variability in housing and handling animals, such as animal acclimatization and recovery time after experiments. Furthermore, there are differences in software (settings) and hardware. By standardization and unification of research methods, the data reliability can be improved, and, ultimately, data from different studies can be compared directly [14].

Recently, we performed an analysis of research characterizing changes in ABR profile in various rat models of tinnitus [15]. We have found a high level of outcome heterogeneity in these studies and have determined that the methodological approach was the primary source of these variations. Therefore, in the present study, we examined factors that could impact the outcome of ABR recordings, focusing on determining if and how the auditory researchers report them. We have surveyed research groups that recently published an assessment of ABR in rats in peer-reviewed journals, collected and analyzed data on variables that could influence the ABR outcome, and compiled recommendations for reporting of ABR research. Our current study intended to improve understanding of possible outcome differences and enhance the quality of data reporting in auditory research.

## 2. Materials and Methods

We have identified research groups that published studies on ABR in rats in the past five years. The corresponding authors were contacted and asked to complete a questionnaire to obtain a detailed experimental protocol. We have identified several discrete differences in the practical approaches based on the answers submitted by the international research groups.

### 2.1. Search Strategy

Two electronic databases, PubMed and Web of Science, were searched for relevant studies. The search was restricted to manuscripts written in the English language. The time frame of publications was set between 2017 and May 2021. The keywords included the following combination of MESH terms:

- Auditory evoked potential” AND “rat”;
- Auditory brainstem response” AND “rat”.

Full-text articles were downloaded when the title, abstract, or keywords suggested that the study was eligible for this research.

### 2.2. Study Selection

Upon preliminary inclusion, studies were read in their entirety to confirm that the inclusion criteria were met.

Inclusion criteria

- Article published in the last five years
- Original research
- Use of rats
- Use of acoustically evoked auditory brainstem responses

Exclusion criteria

- Full text not available
- Literature review
- Lack of information about the experimental group
- Use of electrically evoked auditory brainstem responses

**Figure 1:**
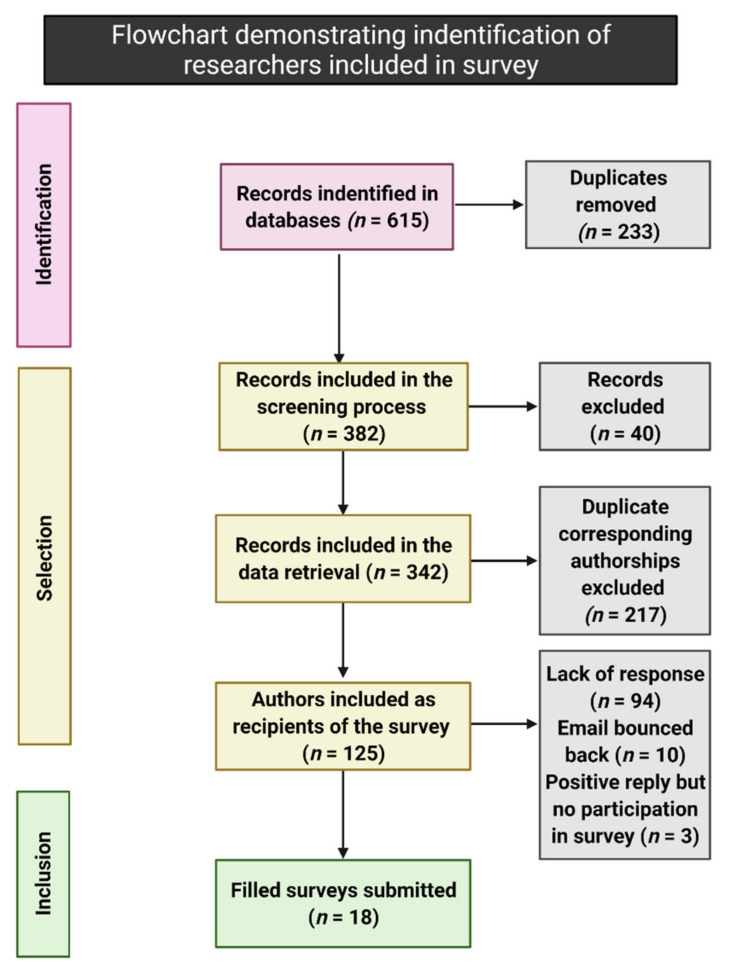
The diagram visualizes the selection process of research groups and researchers invited to participate in the survey. “*n*” signifies the number of records.

Six hundred fifteen manuscripts met the inclusion criteria (Figure 1). After the duplicate removal, 125 publications included were identified, and the names and email addresses of corresponding authors were extracted. The authors have been contacted by email. In the letter, the goals of this study were explained, and collaboration was solicited. A questionnaire (Supplementary Table S1) was appended to be filled and returned upon agreeing to collaborate.

### 2.3. Survey

The survey contained questions concerning three main domains with 13 sub-domains, some of which were earlier identified as significant for rat hearing abilities [15], whereas others were added based on our practical experience. The main domains included animal-related factors, equipment-related factors, and experimental design-dependent factors. The particular factors comprised:

1. Animals used (strain, gender, age)
2. Animal housing
3. Transfer of rats to the ABR experimental room
4. The system used to measure ABR
5. Time of ABR measurement
6. Anesthesia
7. Tympanic membrane evaluation
8. Experimental area
  - experimental room temperature (RT)
9. Animal’s body temperature
  - monitoring of the body temperature
10. Experimental design
11. Parameters measured using ABR
12. ABR acquisition characteristics
13. Stimulus characteristics

Eighteen laboratories worldwide consented and returned the filled survey (summarized in Supplementary Table S2). The obtained information was extracted, analyzed, and synthesized.

## 3. Results

### 3.1. Animals Used

The surveyed groups reported using Wistar, Sprague-Dawley, Long-Evans, Lewis, and Fischer 344 rats (Table 1). One group has not provided information about the strain used. Also, the gender and age of the experimental animals differed.

### 3.2. Animal Housing

#### 3.2.1. Animal Facility

Most of the submitted surveys reported keeping the rats in the animal facility after purchase (14 protocols). In two protocols, the animal facility was a part of the laboratory. Two groups reported holding the animals directly in the laboratory after arrival.

#### 3.2.2. Day/Night Cycle

In all submitted protocols, a conventional day/night cycle (12/12) was maintained.

#### 3.2.3. The Temperature in the Facility

Nine groups reported that temperature varied between 18–23 °C. A single group reported that the temperature varied between 20–25 °C.

#### 3.2.4. Humidity in the Facility

Humidity varied between 30% and 70%. Eight protocols did not provide this information.

#### 3.2.5. The Cage Type and Number of Rats per Cage

**Figure 2:**
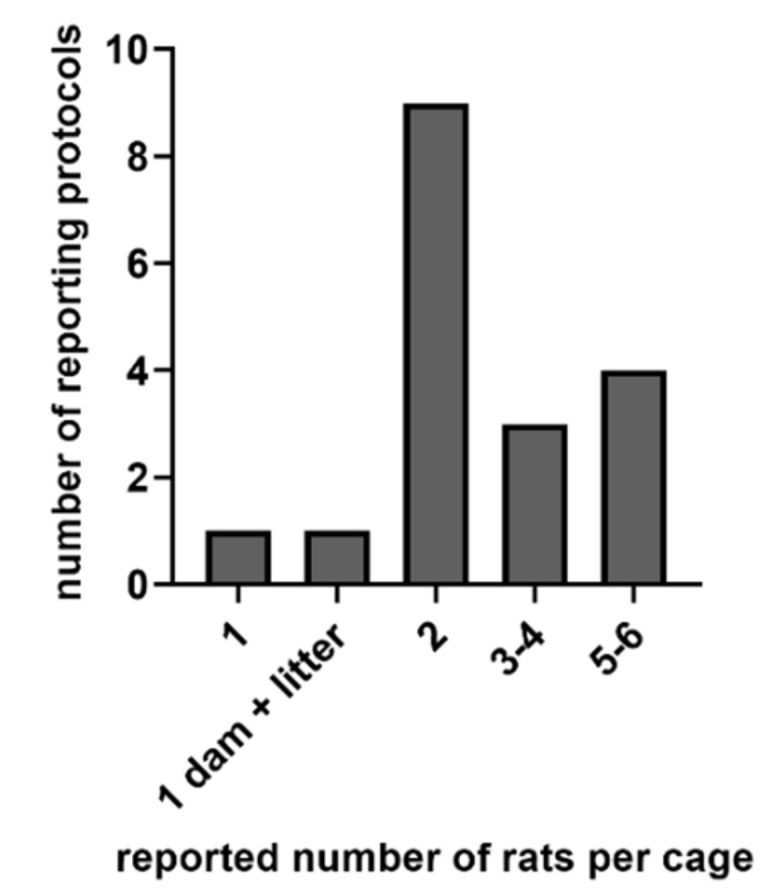
The number of rats per home cage housed in the animal facility, as reported by the researchers in the survey.

Various numbers of rats per home cage were reported. In nine protocols, keeping two animals per cage was reported. In contrast, four protocols reported housing 5–6 rats per cage; in three protocols, 3–4 rats per cage were mentioned, and one protocol reported housing a single animal as well as housing one dam with litter (Figure 2).

In addition to the different numbers of housed rats per cage, the size of cages used varied. According to two protocols, pair-housed rats were kept in the individually ventilated (IVC) cage (size not reported). One protocol reported using 30 × 20 cm cage for two rats; another protocol mentioned using 26 × 40 × 18 cm cage; in two protocols cages 44 × 22.5 × 20.5 cm were used, and in another two, 50 × 31 × 23 cm. Cages of size 60 × 25 × 25 cm or 80 × 60 × 50 cm were used for housing of 3–4 rats. Finally, one group used a 50 × 50 cm cage to house five rats. In five protocols, the designation “standard rat’s cage” was used. Two protocols did not provide this information.

#### 3.2.6. Acclimatization Time

There were differences in the acclimatization time after the arrival from the breeder to the animal facility. In most collected protocols, the acclimatization time was seven days (7 protocols), but four reported 3–4 days of acclimatization. Three weeks of acclimatization were used in one case when the day-night cycle was reversed. In three protocols, rats were bred and/or born in the animal facility. In one protocol, acclimatization was not required. One protocol did not provide information on acclimatization time.

### 3.3. Transfer of Rats to the ABR Experimental Room

In 9 submitted protocols, rats were transferred to the examination room in their home cage. Using a new cage for transfer was reported by seven protocols. Two protocols did not provide that information. In one protocol, the home cage was used to transfer animals, but a new and clean cage was used after recovery from anesthesia.

In 14 protocols, 1–4 rats were transferred simultaneously to the ABR examination room in one cage. Two protocols reported moving 6 and 10 rats in one cage to perform ABR. Additionally, one litter with 15 pups in a cage was transferred to perform ABR. One protocol did not provide information about the number of rats transferred to the ABR room in one cage.

According to nine protocols, rats were acclimatized to the experimental room before ABR; however, the acclimatization time varied from 10–15 min (four protocols), 30 min (three protocols) up to 1 h (two protocols). One protocol reported bringing fully anesthetized rats to the ABR room. In one protocol, rats were transported to the laboratory daily for few consecutive days before performing ABR measurement. Eight protocols reported lack of acclimatization period. One protocol did not provide the information.

Assessing the stress level was reported only by three research groups. The following parameters were used as indicators of perceived stress: abnormal posture, changes in motoric activity, reduced water and food intake, gait disturbance, erected hairs, weight loss, hypersalivation, abnormal licking, chewing movement, tremor, desensitization, moaning, and aggressive behavior. For the rodent pain assessment, The Rat Grimace Scale (RGS) was used in one protocol. The RGS assesses changes in four action domains: orbital tightening, nose/cheek appearance, ear and whisker positions [16].

### 3.4. The System Used to Measure ABR

Two commercially available systems—Tucker Davis Technologies (TDT, Miami, FL, USA) and Intelligent Hearing Systems (IHS, Miami, FL, USA)—were predominantly used to record ABR. Using the TDT system was reported by eleven submitted protocols, whereas IHS by four protocols. In 3 protocols, other instruments, namely Neuro-Audio 0710 (Ivanovo, Russia), Interacoustic (Middelfart, Denmark), and ADInstruments (Castle Hill, Australia), were mentioned.

The year of purchase differed between selected protocols. While the experimental groups purchased the TDT system between 2009 and 2019, the IHS system was acquired between 2013 and 2016. Four protocols did not provide information about the system used for electrophysiological recording or the year of purchase.

### 3.5. Time of ABR Measurement

Fourteen groups reported performing the ABR measurement always at the same time of the day. In most of those protocols, rats were tested in human morning times (rat’s dark phase). When the day’s recording time was not strictly controlled, it varied between 8 a.m. and 7 p.m. Time of measurement was strictly linked to the number of tested rats per day.

### 3.6. Anesthesia

During ABR recording, rats need to be anesthetized to prevent movements. The anesthetics used varied between the protocols. The majority used the combination of ketamine and xylazine administered in a weight-dependent manner (mg/kg, body weight, BW). In 12 protocols using ketamine/xylazine, the drugs ratio differed between 3:1; 4.5:1; 6:1; 6.6:1; 8.5:1; 9:1; 10:1; 11:1; 20:1; 28:1 and 30:1. Isoflurane (inhalation) was reported in three protocols (4–5% to induce, 1.5–2% to maintain), and sodium pentobarbital (50 mg/kg) in one protocol. Using the mixture of ketamine (75 mg/kg) and medetomidine (Domitor) (0.3 mg/kg) was reported in 1 protocol.

The injectable anesthetics may be administrated by intraperitoneal, intramuscular, or intravenous injection [17]. In most submitted protocols, intraperitoneal (six protocols) or intramuscular (five protocols) injections were used. The initial anesthesia was reported in two protocols by intraperitoneal injection and supplemental dose (1/2 of the initial dose) as an intramuscular injection. The subcutaneous application was used in two protocols.

Different reflex-based methods were used to check the depth of anesthesia (Table 2). The majority of the research groups checked more than one reflex to determined the depth of anesthesia.

A common side-effect of general anesthesia in mammals is the disrupted thermoregulation. Nine protocols reported monitoring of body temperature and breathing of the anesthetized rats. The mucosal membrane color and heart rate assessment were reported in three and two protocols, respectively (Table 3).

In nine protocols, the researchers were covering the eyes of animals during the ABR measurement. Covering the eyes prevents a corneal abrasion and suppresses the animal’s blink reflex, which might be a source of ABR artifacts. In one submitted protocol, rat’s eyelids were closed when using isoflurane anesthesia, or a layer of ophthalmic ointment was applied to the eyes.

### 3.7. Tympanic Membrane Evaluation

Twelve protocols reported performing otoscopy. In ten of them, earwax was removed if needed.

### 3.8. Experimental Area

The size of the experimental room/chamber differed. The majority of groups reported room sizes varying between 3 m^2^ and 150 m^2^. Three groups have used a room that ranged in size between 1 and 9.66 m^2^; three other groups used rooms between 10 and 20 m^2^ in size and another four, 30 to 40 m^2^. Two groups used the ABR-laboratories of a size between 64 m^2^ and 150 m^2^. Five groups provided the size of ABR chambers, which varied between 0.000182 m^2^ and 5 m^2^. Using a Faraday cage was reported by eight respondents.

#### Experimental Room Temperature

Nine groups reported maintaining a stable temperature in the experimental room. The temperature varied between 19 and 23 °C (6 groups). Two and one groups reported higher room temperatures (24–25 °C and 37 °C), respectively. Nine groups reported not measuring the temperature in the experimental room.

### 3.9. Animal’s Body Temperature

Maintaining a stable body temperature of rats was reported by 15 respondents. Using a heating pad was reported in 9 protocols, non-heating pads were reported in six protocols.

#### Methods for Monitoring of the Body Temperature

Rectal thermometers (6 protocols) or electronic thermometers (5 protocols) were used to measure the rat’s body temperature. In one protocol, the temperature was measured with an infrared thermometer every 5 min. One protocol highlighted that using a rectal thermometer is not applicable for pups. Therefore, the contact area temperature between the heating pad and the pup’s body was measured.

### 3.10. Experimental Design

**Figure 3:**
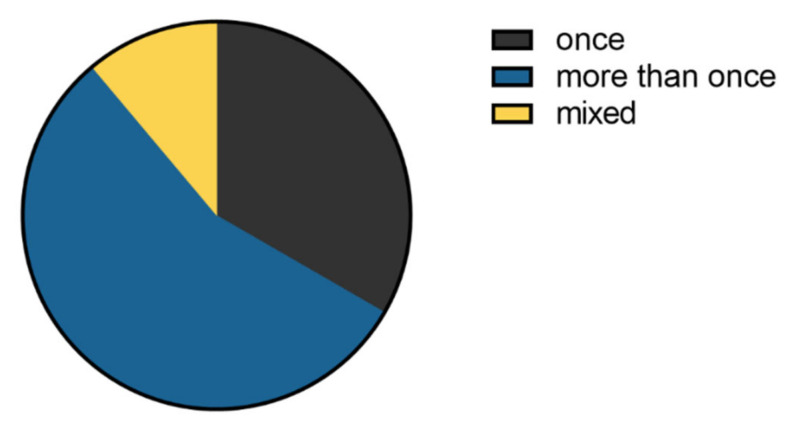
Number of ABR recording sessions per experimental animal as reported in the survey. More than half of the 18 research groups reported using the same animal more than once for the ABR recording.

The number of times the same animal was subjected to ABR recordings varied (Figure 3).

For the repeated measurements, a critical factor is a post-ABR treatment (after anesthesia), which impacts the rat’s well-being and the process of posterior electrophysiological recordings. According to almost all collected surveys, rats were housed individually in a clean cage lined with paper towels instead of bedding to prevent choking. The cages were kept warm until rats became responsive after anesthesia (whiskers twitching and beginning to move around) and could move freely (about one hour). After that, the animals were returned to their home cage but separated from the other rats (9 protocols). A single protocol reported that rats were placed in a new, clean cage and housed there until the subsequent ABR measurement.

### 3.11. Parameters Measured Using ABR

In the majority of protocols, the ABR was used to estimate hearing thresholds (17 protocols). Analysis of amplitude and latency was reported by 15 and 14 protocols, respectively. When ABR was used to measure hearing thresholds, the number of ABR recordings varied. Two or more recordings around the threshold measured were reported by seven and three protocols, respectively. Estimating the hearing thresholds based on a single recording was indicated in 6 protocols.

### 3.12. ABR Acquisition Characteristics

#### 3.12.1. Signal Delivery/Speaker Placement

In the submitted protocols, sound stimuli were reported to be presented directly to the ear canal (thirteen protocols) or the free field (five protocols). The distance between the speaker and ear varied between 2 and 10 cm.

#### 3.12.2. Electrodes

Most of the research groups reported using three stainless-steel electrodes to record electrophysiological responses (17 protocols). In two of them, electrodes were surgically fixed. One protocol has reported using four electrodes.

The electrodes were inserted subcutaneously: one on the mastoid of the tested ear (reference), one on the vertex (active), and one on the contralateral mastoid (ground) reported in 16, 18, and 6 protocols, respectively. Some protocols reported locating the ground electrode on the back of the animal (four protocols), hind paw (three protocols), neck (one protocol), or occiput (one protocol). Two protocols did not provide information about electrode placement.

Electrode impedance ranged between 0.3 and 5 kΩ. The impedance between 0.3 and 1 kΩ was reported in five protocols; between 1 and 3 kΩ in four protocols; <5 kΩ in one protocol. Three groups have not measured the impedance, and five have not provided information about the impedance.

The electrodes were purchased from various manufactures. Five groups used Rochester Elektro-Medical, (Lutz, FL, USA). Other manufacturers were: Grass Instruments (West Warwick, RI, USA); Technomed (Plymouth, MN, USA); Medtronic (Dublin, Ireland); Natus Ultra (Pleasanton, CA, USA); IHS (Miami, FL, USA) and TDT (Miami, FL, USA). Two groups reported using electrodes from an unknown source. Four groups have not provided information about the manufacturer of the electrodes.

The usage time of the electrodes varied between submitted protocols. In four protocols, the same electrodes were used between three and five times; five groups used the same electrode six to ten times and up to twenty times (one group). Usage of the electrodes “until dull” was reported in three protocols. The electrode’s quality was assessed by measuring impedance and by visual inspection (stereo microscope). In two protocols, electrodes were surgically fixed. In one protocol, the usage time (maximum one hour) was reported instead of the number of ABR recordings. Three protocols did not provide information about the usage time of the electrodes. Ethanol (70–75%) or Clidox (a chlorine dioxide-based sterilant) were used for disinfection.

### 3.13. Stimulus Characteristics

#### 3.13.1. Tested Frequencies and Intensity Range

ABR responses were elicited by tone burst (9 protocols) and click (3 protocols). In five protocols, both the click and tone burst were applied. The 5 ms tone burst was used (nine protocols). In two protocols, 1 ms and 1000 ms sounds were applied. Three protocols did not specify the length of the signal. The number of cycles was 2-1-2 (three protocols); 1-3-1; 0.5-4-0.5 or 0.5-0-0.5 (each used once). Six protocols did not provide information about the number of cycles.

Sound stimuli were delivered in the frequencies of 0.5–32 kHz (two protocols); 1–64 kHz (one protocol); 2–32 kHz (one protocol); 2–48 kHz (one protocol); 4–32 kHz (two protocols); 5–11.4 kHz (one protocol); 5–40 kHz (one protocol); 8–16 kHz (one protocol); 8–32 kHz (three protocols) and 10–32 kHz (two protocols).

The sound intensity levels gradually decreased from 100 dB SPL in 5–20 decrements (six protocols). In five protocols, 80 dB SPL was the maximal intensity used. In one protocol, only a single intensity was used (e.i. 60 dB).

#### 3.13.2. Repetition Rate/Stimulus Rate

Sound stimuli were presented at a rate of 11 (one protocol); 19 (one protocol); 20.2 (two protocols); 21 (seven protocols); 37 (one protocol) bursts/s.

#### 3.13.3. Polarity

The majority of ABRs were recorded in the alternation polarity (eight protocols). Rarefaction and condensation polarities were reported in two protocols. Six protocols did not provide polarity information.

#### 3.13.4. Number of Averages and Analysis Time

Original data are averaged to achieve an increase in the signal-to-noise ratio. The number of averages varied between 300 and 2000. In the majority of reports, it was 500–512 (seven studies). Others used 100–1024 averages (four protocols) or 300 and 2000 (two protocols). One protocol did not provide this information. The period of analysis time in the submitted protocols was 10 ms (eight protocols). 20 ms was reported in one protocol. Eight protocols did not provide that information. Only half of the collected protocols provided the sampling rate information. In the majority, 25 kHz was applied (five protocols).

#### 3.13.5. Filters

Averaged ABR waveforms were bandpass-filtered between 30 Hz up to 1.5–2 kHz (two protocols); 30 Hz–3 kHz (two protocols); 300 Hz–3 kHz (seven protocols) and 100 Hz–3 kHz (two protocols). Using of a notch filter was reported in five protocols. It varied between 50 and 60 Hz.

#### 3.13.6. Click ABR

Click was used to elicit ABR responses in eight submitted protocols. In the majority of protocols, the click duration was 0.1 ms. In two protocols, a prolonged stimulation (5 and 10 ms) was applied. Various intensity levels were used in collection protocols. In three protocols, only single intensities were used (50 and 70 dB SPL). In other protocols, the sound intensity levels gradually decreased from 100 dB SPL in 5–10 decrements. Sound stimuli were presented at a rate of 11, 21, 31, and 100 bursts/s. Three of the submitted protocols did not provide information about the repetition rate. Alternating polarity was reported in five protocols. The sampling rate used by one group was 100 kHz and 25 kHz by another group. Averaged ABR waveforms were bandpass-filtered between 30 Hz–3 kHz (one protocol), 30 Hz–1 kHz (one protocol), and 300 Hz and 30 kHz (three protocols). Usage of the notch filters was reported in two protocols.

## 4. Discussion

The present work aimed to analyze factors that could influence the outcome of ABR in rats and, therefore, should be reported in published research. The collaboration with researchers performing ABR in rats resulted in positive feedback from 18 corresponding authors. We have identified numerous dissimilarities in the experimental procedures and assembled reporting recommendations regarding specific domains based on the researchers’ input and the current literature.

**Figure 4:**
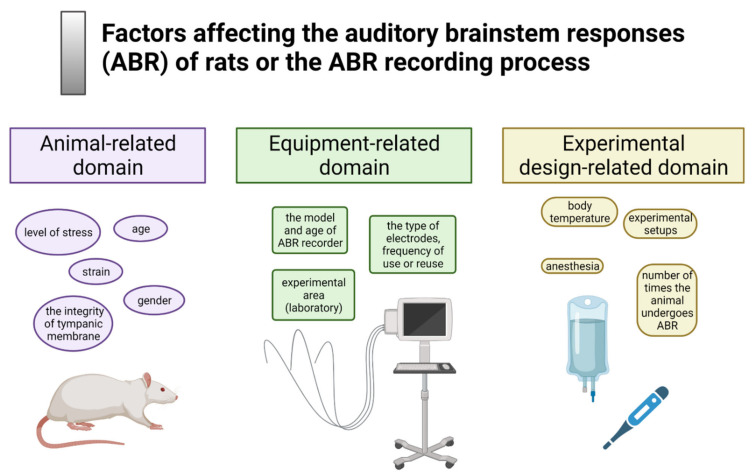
The survey examined three domains reflecting animal-, equipment-, and experiment-dependent factors influencing the ABR outcome. Created with BioRender.com (accessed on 28 November 2021).

The factors possibly influencing the ABR outcome were grouped into three domains: animal-dependent, equipment-dependent, and experiment-dependent (Figure 4). The animal-dependent domain comprised factors known to affect rats’ hearing, such as strain, gender, and age of animals. In addition, factors indirectly influencing the hearing thresholds were considered and included all situations generating stress (emotional and social), such as animal handling, transport, transfer, the type or size of the home cage, the number of animals per home cage, and acclimatization period after delivery from the breeder.

The second domain included factors related to the ABR equipment: the type and production year of the ABR recorder, the type of electrodes used, and the experimental area.

The third domain encompassed all variables that depend on experimental protocol, such as the type and route of anesthesia administration or the number of times the ABR measurement was performed.

The influences of factors belonging to the three domains on the ABR outcome are discussed below.

### 4.1. Animals Used

Different rat strains (of both genders) are used in ABR-related research. The age of animals varied from 1 to 24 months. The strain- and gender-dependent effects on electrophysiological recordings in rats were observed in many studies. Young adult Sprague-Dawley rats have lower ABR thresholds at 2, 4, and 8 kHz than Long-Evans rats (frequencies tested 2, 4, and 8 kHz). Moreover, Sprague-Dawley rats’ latencies (I–IV waves) are shorter and amplitudes larger than Long-Evans rats [18]. Fischer 344 rats have better hearing abilities (about 20 dB) than Brown Norway rats but only in low frequencies (around 4 kHz). In contrast, Fischer 344 rats have a higher ABR threshold than FBN rats in high frequencies (32 kHz) [19].

There are gender-dependent effects determined in Long-Evans, Sprague-Dawley, Fischer 344, and Dark Agouti rats. Young female Long-Evans rats (1–2 month-old) have better hearing abilities than the male rats. Significantly higher thresholds were observed in male rats at both low (1 and 4 kHz) and high (32 and 42 kHz) frequencies (tested frequencies: 1–64 kHz) [20].

In Sprague-Dawley female and male rats, the amplitude and latency had not differed when the rats were younger than 70 days old [21]. However, in older animals, differences were observed. Female Sprague-Dawley rats (2.5–8 month-old) had shorter ABR latencies (wave I–III) than the male rats. The gender effect on latency of wave IV was observed only when a low repetition rate (8/s) was used [22]. The amplitude of ABR waves of the female rats was more prominent than the male Fischer 344 rats. The most noticeable difference was detected in the amplitude of wave I. The differences in hearing thresholds of Fischer 344 rats were seen in 12 month-old rats. Aged male rats had higher average hearing thresholds (at 4–32 kHz; frequencies tested: 2–40 kHz). The aged-related hearing loss was already detected in 3 month-old male rats, whereas in female animals, first changes were observed in 8 month-old animals [23]. Gender-dependent changes in the ABR profile were also confirmed in 18 month-old Dark Agouti rats [24].

The differences mentioned above could be linked to estrogen receptors expressed in the auditory system. In agreement with that, various studies demonstrated an association between the menstrual cycle and hearing threshold [25,26]. Generally, a higher level of endogenous estrogen was associated with a better hearing function [27]. Due to that, in female rats older than five weeks, the estrous cycle must be considered during ABR analyses [28]. Importantly, sexual maturation might be reached much later in other rat strains (<50 days old) [29]. The estrous cycle may also alter the anesthetic susceptibility of the animals [30]. In males, the age of sexual maturity varies between individual rats [31]. Therefore, the weight of an animal might not be an accurate indicator of its age. Also, differences in the body weights and animal age between different colonies from the same supplier as well as between suppliers should be considered.

Moreover, the body mass and tissue mass in the head/neck region can differ between the animals, thus, impacting the quality of measurements (discussed in detail in Section 4.12.2).

- **Reporting recommendation:** Include gender and age of animals in the study report.

### 4.2. Animal Housing

A critical factor impacting the hearing abilities of animals is the environmental sound level in the animal facility. It has been reported that the primary source of noise is human activity [32,33]. The noise level was reduced during after-work hours.

Additionally, it is supposed that the susceptibility to noise exposure is strain and age-dependent. Exposure to noise during the light phase can result in disturbances in a rat’s sleep pattern. The detrimental effect of sleep deprivation on hearing abilities has been described in both mice and rats. Female Balb/C mice subjected to sleep deprivation for both one day and segmental five days (sleep deprivation for 22 h and two-hour sleep-free) demonstrated a temporary increase in the amplitude of ABR wave I, but not in the thresholds. The amplitude returned to the baseline level two weeks later [34]. Male Wistar rats subjected to sleep deprivation for nine days had higher hearing thresholds at 8, 16, and 32 kHz, but not at low frequencies (2–4 kHz). Amplitudes and latencies were not assessed [35]. Differences in sleep patterns were also observed between rats depending on enhancing cage complexity. Male Wistar rats housed in enrichment cages slept longer than rats housed without enrichment. Cage complexity alters the level of agonistic behavior in rats, as well [36].

The noise-generating activities inside the animal facility should be reduced to the minimum [32]. Nevertheless, there is still a need for a universal guideline for safe noise exposure in animal facilities.

Not only noise but also differences in the temperature and humidity in the animal facility can affect hearing abilities in rats. The information in the submitted survey provided insights about various temperatures in the facilities (between 18–26 °C) and humidity (between 35% and 70%). Both parameters are involved in the capacity for thermoregulation and the transmission of pathogens [37]. While high humidity can enhance the proliferation of bacteria and facilitate the release of ammonia in animal cages, low humidity can induce changes in the middle ear. Sprague-Dawley rats housed for five days in a low humidity environment (10–12%) had a higher incidence of serous effusions in the middle ear than the control group (humidity 50–55%). Nevertheless, the changes in the middle ear were temporary [38].

- **Reporting recommendation:** The level of noise and the temperature and humidity in the animal facility should be reported.

#### 4.2.1. The Cage Type and Number of Rats per Cage

Housing conditions affect the behavioral and biological responses of animals in a gender-dependent manner. While male Wistar rats were more affected by crowding stress, corticosterone level in plasma of female rats was higher when animals were single-housed (experiment duration: eight consecutive days) [39]. Minimum space allowances for laboratory rats are legislated based on weight and stocking rates. During the estimation of the optimal cage space for rats, two factors should be considered: the number of rats per cage (group size) and the physical floor space available to each rat (cm^2^/animal) [40]. Increasing housing density is associated with crowding stress [41].

- Reporting recommendation: Report the housing conditions in the Supplementary Materials.

#### 4.2.2. Acclimatization Time

The crucial factors in animal studies are transport and acclimatization. The experiments should be carried out after the adaptation of the animals to the new environment to avoid stress, which is known to influence the hearing thresholds [42]. Interestingly, the acclimatization time indicated in the submitted survey varied between the research groups. Short acclimatization periods affect the reliability of research results and hurt animal welfare [43]. Already five hours of transport (during the dark phase) evoked changes in rat heart rate and body weight but not in the body temperature [44]. Moreover, rats transported for 5 h required at least three days of acclimatization to the new place. In addition, if any shift in the light-dark phase occurs during transport, the period of acclimatization should be extended [44]. Also, age and health status can have an impact on stress occurring during transport [45]. A one-week of acclimatization is recommended for all animals to prevent the development of stress-induced conditions. Reversing the day/night cycle in animals extends the acclimatization time.

Before performing experiments, rats should be accustomed to the researchers and experimental procedures [46]. Although animals do not readily habituate to the handling, such habituation may help to reduce the stress response [47,48].

- **Reporting recommendation:** Indicate an acclimatization period in the protocol.

### 4.3. Transfer to the ABR Experimental Room

Behavioral changes are known to occur when animals are moved to a new facility, a different room, and moved around within the same room [49]. An additional source of stress for rodents can be cage change [50]. Therefore, all these factors should be taken into account when planning experimental procedures. Physiological (e.g., blood pressure, stress hormone levels, body weight) and behavioral changes may arise from changes in the immediate environment of the animals, e.g., changes of or inside the cage [51]. Interestingly, the serum corticosterone concentration of mice changed after handling and transfer to a clean or dirty environment. In addition, a significant corticosterone increase was detected 15 min after cage change and returned to baseline level 60 min after cage change [52]. Therefore, cage cleaning should not be performed on the same day when scheduled experimental procedures and the ABR recordings. A difference in rat behavior was also observed when the animal is familiar with the staff involved in activities [53].

The number of rats per cage during transfer to the experimental area from the animal facility depends on the experimental design. Nevertheless, mixing rats from different cages or the transfer of single animals should be avoided. Acclimatization to the new environment should be allowed. The length of the acclimatization depends on individual animals, and as long as they appear to be stressed, experimental procedures should not be performed. As for the very young animals, it is assumed that keeping the litter together is less stressful than separating the pups for testing.

The level of stress experienced by the experimental animals should be taken into account in data analysis. Using stressed animals might result in considerable and unintended effects on research data [54]. To estimate stress and pain level in rats, the Rat Grimace Scale can be used. Additional information is available in the Recognition and Alleviation of Pain report in *Laboratory Animals* [55]. When the animal facility and the experimental room are located in separate buildings, the outside temperature’s effect during animal transfer should be considered.

- **Reporting recommendation:** Report the details concerning animal transfer from the animal facility to the experimental area. Indicate the time of the last cage cleaning.

### 4.4. The System Used to Measure ABR

In the submitted surveys, various instruments were reported to measure the ABR. Unfortunately, no published studies compared ABR recordings performed in the same animals but with different equipment. Nevertheless, there is a possibility that the results will vary when using diverse ABR equipment.

Other factors related to the equipment are the distance between the speaker and the ear and the manufacturer of electrodes. Therefore, future studies are needed to exclude the impact of equipment on ABR outcomes. It is essential to check the system and calibrate stimulation frequencies on each day of recording before animals are anesthetized [56].

- **Reporting recommendation:** Report the type of system, conditions, and consumables used to measure ABR.

### 4.5. Time of ABR Measurement

To minimize the possible time-of-day effects, all testing should be performed at the same time of the day. Previous studies have shown the impact of circadian rhythm on response to acoustic stimulation [57]. In addition, the noxious effect of noise varies at different daytimes. Mice exposed to noise trauma (6–12 kHz, 100 dB SPL for 1 h) in the morning recovered two weeks after exposure, whereas mice subjected to noise in the night had elevated ABR thresholds two weeks after stimulation (around 10–20 dB) [58]. Nevertheless, hearing loss, hair cell loss, and changes in the central auditory system are less predictable when using broad-band noise than narrow-band noise [59]. Future studies should determine if changes in light-dark cycles lead to fluctuations in ABR profiles.

- **Reporting recommendation:** Indicate using light/night cycle, length of each phase, and time of the phase switch.

### 4.6. Anesthesia

One of the limitations in ABR studies is a difference in anesthetic drugs used, also observed in the submitted surveys [60]. Age, sex, genetic and environmental factors, and inherent interindividual differences contribute to the anesthetic susceptibility of rodents [61].

The hearing thresholds of adult male Long-Evans rats increased after administration of isoflurane (1.5–2%; 4%) in comparison to animals anesthetized with a mixture of ketamine and xylazine (50 mg/kg+ 9 mg/kg) [62]. Even though female Long-Evans rats were anesthetized a few times with ketamine+ xylazine, the effect of drugs on ABR amplitudes and latencies was not significant [60].

Although the effect of ketamine on ABR recordings is minor, rats anesthetized with this drug are more likely to develop corneal lesions than rats anesthetized with isoflurane, which is essential for long-term studies. In addition, the susceptibility to develop corneal injury is strain-dependent. Wistar, Long-Evans, and Fischer 344 rats have a greater incidence rate of corneal lesions after anesthesia than Sprague-Dawley rats [63]. Covering animals’ eyes during anesthesia reduces the risk of developing a corneal injury, and thus, should become standard practice.

Despite using the same anesthetics, different amounts of drugs are often applied, possibly impacting cardiac function [64]. Also, various combinations of ketamine and xylazine affect the anesthetic conditions (working time and depth of anesthesia) [65]. It is assumed that different drugs ratio might reflect the recommendations of the local Animal Welfare Office.

The route of anesthetics delivery is also of importance. Although the majority of studies used intraperitoneal injections, intramuscular injections might be more beneficial for animals. Inappropriate restraint during intraperitoneal injections can lead to multiple injections, which might harm the recovery process [66]. Beginners should practice proper restraint techniques. Refer to video tutorials for the restraining and injections method [67].

Different methods are being used to assess the depth of anesthesia. Improper assessment of anesthesia depth might result in recording artifacts and low amplitude of ABR waves.

- **Reporting recommendation:** Report the dosage of anesthetics and the route of administration.

### 4.7. Tympanic Membrane Evaluation

The integrity of the tympanic membrane is a crucial step before the ABR recording. Before ABR thresholds determination, otoscopy should be performed. The animals with abnormalities in the ear canal or tympanic membrane should be excluded from further experiments.

- **Reporting recommendation:** Report assessing (or not) the ear canal and tympanic membrane.

### 4.8. Experimental Area

The experimental area should be a sound-proof, floating booth explicitly designed for auditory physiology and resistant to building/room vibrations. The booth should also be grounded to act as a Faraday cage. Any cables, noise generators might generate unwanted electrical noise. Therefore, to determine the noise floor, a saline test should be conducted as a part of the calibration process. During that test, electrodes are submerged in saline. The electrical recordings that are higher than 100 nV suggest that the level of noise should be reduced.

In the submitted protocols, no differences were reported between temperature in the animal facility and experimental room. However, the term “room temperature” is somewhat flexible and might mean different temperatures, and the fluctuations in RT should be considered a confounding factor [68]. When air-conditioning does not control the temperature, RT might fluctuate depending on the geographical location and seasonal changes. As a result, conducting experiments in temperature-uncontrolled conditions might affect the outcome reproducibility [68]. Due to that, the room temperature should be measured and reported.

- **Reporting recommendation:** Report the use (or lack of) of the Faraday cage and the actual temperature in the experimental room.

### 4.9. Animal’s Body Temperature

Changes in animal’s body temperature have an impact on the experimental outcome. Decreasing body temperature prolongs the recovery period after anesthesia [60]. In addition, a decrease of body temperature by 0.5 °C or more may significantly alter ABR latencies and amplitudes [69]. Because of that, rats’ body temperature should be maintained at about 37 °C and should not vary more than 0.4 °C [70]. Although the rectal thermometer is recommended to control body temperature, the relationship between rectal and core temperature is still unclear. Thermometer insertion depth has a considerable effect on the accuracy of measured temperatures [71]. A report about a heating pad (surface temperature of 37.2–38.9 °C) demonstrated effectively raising the rectal temperature and maintaining the rat’s core temperature for 60 min anesthetic period [72]. Importantly, using a rectal thermometer with an electrical pad inside the chamber might be a source of noise and impact on ABR recordings. Using a non-electrical heating pad might be a more beneficial solution.

- **Reporting recommendation:** Report if and how the body temperature of animals was maintained and measured during the ABR recording.

### 4.10. Experimental Design

The submitted surveys reported varying numbers of ABR recordings per animal and different recovery times between consecutive recordings. The anesthesia might impair thermoregulation, leading to core-to-peripheral blood redistribution. As a result, the inner ear’s efficiency could be altered. However, hypothermia can be reduced by pre-warming the animal before anesthesia [73,74]. Post-operative care should include observation of food intake and feeding behaviors of rodents [75]. What is essential, placing a rat in a new cage after recovery from anesthesia is an additional stressor and should be avoided. The introduction of changes in the immediate environment of experimental animals should be performed in animal groups to prevent or lessen emotional stress. That also applies to the transfer to the testing room [44].

- **Reporting recommendation:** Report the number of ABR recordings per animal and the recovery time between consecutive recordings.

### 4.11. Parameters Measured Using ABR

A threshold is defined as the lowest intensity required to produce a reliable ABR waveform. In the literature, different components are considered as ABR thresholds. Some research groups use the responses of II–III and IV–V waves, whereas others use wave II or wave IV to detect hearing thresholds [76]. Nevertheless, in rats, wave II is the most prominent, and the last disappearing when the sound intensity is decreased. Due to that, we recommend using wave II to estimate the threshold. Estimating the ABR threshold is a subjective procedure because multiple ABR traces (2–3) must be collected at a near-threshold intensity to recognize the threshold properly. Nevertheless, noise and artifacts can mask small threshold responses and obstruct correct analyses.

Latencies and amplitudes are used to detect hearing abnormalities. Both parameters are sensitive to changes in body temperature, electrode placement, and sound intensity levels. The extension of ABR latencies and the decrease in ABR amplitudes can be observed when stimulus intensities are decreased [22]. Also, strain-specific differences should be considered.

- **Reporting recommendation:** Indicate the number of the ABR traces at near-threshold intensity.

### 4.12. ABR Acquisition Characteristics

#### 4.12.1. Signal Delivery/Speaker Placement

The speaker placement might negatively impact ABR recordings since the external ear or pinna amplify or attenuate sound. The impact of an animal’s pinna position on hearing has been confirmed in reindeer. Depending on the position of animals’ external ear and the distance from the speaker, a different threshold was detected (difference range about 21 dB) [77]. The speaker should be placed in the same plane as the tested ear (at an angle to the walls). Putting the tested animal in a natural position is essential: its head should be placed in the same plane as the calibration microscope.

- **Reporting recommendation:** When signal stimulation is not delivered directly to the ear, please report the distance between the speaker and the tested ear.

#### 4.12.2. Electrodes

The majority of the submitted protocols reported using three electrodes placed subdermally to record evoked potentials. An incorrect electrode placement can induce recording noise as well as changes in the ABR waveform. Inconsistent placement of active and ground electrodes leads to a higher amplitude of wave I and lower amplitude of wave V. It results in poorer quality of the ABR waveform and errors in data analysis, mainly hearing threshold. To reduce the impact of electrode placement on the ABR recordings, mark electrode placement on the skin surface if repeated measurements are performed (especially if they are to be compared over time).

Before the ABR recording, electrodes should be sterilized. Lower quality of ABR waveforms (lower artifacts suppression) may arise from the too-high impedance. Nevertheless, the level of maximum acceptable impedance differs between various ABR systems. Generally, impedance should be less than 3 kΩ. Determine the resistance between recording and active electrodes, as well. The usage time depends on the manufacturer of electrodes. Before using electrodes, perform a visual inspection of electrodes under a stereoscope to exclude corrosion, bend of electrodes, or other damage. If the impedance is over 3 kΩ, adjust the electrodes in the scalp and retake the measurement. Continue to manipulate the electrodes until the impedance drops below 3 kΩ [78].

The ABR electrodes should be sterilized shortly before use. The quality of ABR waveforms (lower artifacts suppression) may be affected by a high impedance, resulting from the rats’ body mass and tissue mass in the head/neck region [79,80]. The subcutaneous fat layer has a high electrical resistance and low conductance properties, whereas skin and muscle have low resistance and high conductance. Because of the above factors, achieving low impedance might not be feasible in some cases [81]. Notably, both body mass and muscle mass are sex- and age-dependent [82,83].

- **Reporting recommendation:** Report testing the electrode impedance before ABR; report the result of impedance test.

### 4.13. Stimulus Characteristics

#### 4.13.1. Tested Frequencies and Intensity Range

The hearing range of rats is approximately 250 Hz to 80 kHz, with the most significant sensitivity between 8 and 38 kHz. Despite that, the standard testing range includes frequencies above 4 kHz to 32 kHz [84]; in some studies, only higher frequencies (>8 kHz) are assessed. The hearing range results from the functionality of higher frequencies in rats’ communication. Rats use two main ultrasonic vocalizations frequencies of 22 kHz and 50 kHz [85]. However, the type of vocalization depends on the situation; for instance, in response to discomfort, or physical pain, rats emit audible sound around 4 kHz, described as a squeal [86].

Although a click does not provide frequency-specific information, it might be used to estimate the hearing threshold in rats’ lower frequency range (1–10 kHz) [12]. A single report suggested that click is related to 8 and 10 kHz [87]. The intensity range should be extended when hearing impairment is determined.

Moreover, it is relevant to report the way the sounds are calibrated and monitor the quality of the sound stimuli during the experiment.

- **Reporting recommendation**: Report all frequencies measured.

#### 4.13.2. Repetition Rate/Stimulus Rate

Repetition rate has an impact on amplitude as well as on latency. Nevertheless, the observed changes are age-dependent. In young animals, the peak amplitude decreased when the repetition rate increased [76]. Similar results were obtained when using young Sprague-Dawley rats [22]. Despite changes in repetition rate, wave IV was always clearly visible in young animals. In aged animals, the amplitude was generally decreased, and manipulation of stimulus rate did not differ results [76].

Increasing repetition rate reduces ABR measurement times and shortens anesthesia time in rats [12]. Nevertheless, increasing the repetition rate might decrease the magnitude of the ABR responses [88]. Using the rate of 21/s is recommended to reduce the effects of noise from the 50/60 Hz cycle of mains power. In young Fischer 344 rats (244–412 g), there was no effect of increasing repetition rate on ABR latencies. Nevertheless, increasing repetition rate (from 5/s to 50/s) leads to prolonged latency of wave III-V in aged animals (20–23 months old) [76].

Another critical aspect of ABR measurements is the sequence of stimuli presentation. Research groups report using random or systematic stimuli sequences, but the consequences of the choice remain unclear. The compromise between ABR measurement time and the adaptation of the auditory system to ABR signal stimulation is essential in studies analyzing either amplitude or latency of wave I [89]. One of the solutions to shorten the time required to perform ABR could be using the interleaving tones at different frequencies instead of testing each frequency serially, which was observed in mice and Mongolian gerbils. Notably, similar studies have not yet been performed on rats.

- **Reporting recommendation:** Report the repetition rate and stimulus sequence.

#### 4.13.3. Polarity

The effect of polarity on ABR recordings is discussed in the literature. Although using rarefaction polarity results in higher ABR amplitudes, alternating polarity (switching between condensation and rarefaction) reduces artifact and provides a better visible response. Therefore, the type of polarity depends on the experimental area condition.

- **Reporting recommendation:** Report the type of polarity used.

#### 4.13.4. Number of Averages and Analysis Time

Applying not enough averages results in improper separation of the neuronal activity related to the presentation of auditory stimulus from unrelated activity [90]. Therefore 512 averages are recommended. That number provides a balance between the signal quality and reduction of the time to complete testing. Nevertheless, a higher heterogeneity of ABR results obtained from Wistar rats was reported when 512 averages were used, compared to 64–256 averages [12]. Using a constant number of averages at near-threshold stimulation is recommended. Rats with hearing loss require more averages than rats without hearing impairment. Although the ABR waves in rats can be recorded within 6 ms from the stimulation, 10 ms might be insufficient to record rat’s response since rats with hearing loss require longer analysis time (20–30 ms) than rats without hearing loss.

- **Reporting recommendation:** Indicate the number of averages and the analysis time.

#### 4.13.5. Filters

The function of bandpass filters in electrophysiological studies is to improve the signal-noise ratio and reduce distortion of the ABR waves. Depending on the equipment to record ABR, different parameters are recommended, namely 300 Hz–3 kHz. Notch filters should match the frequency of the mains power coming from the wall. Due to differences in ABR experimental area, different parameters might be used.

- **Reporting recommendation:** Report filters used.

The recommendations on reporting on individual factors possibly influencing the ABR outcomes and reporting methods (main body of the manuscript or Supplementary Information) are summarized in Table 4. Depending on the type of journal, the reporting domains can be included in Materials and Methods and Supplementary Materials or entirely placed as Supplementary Data, as many publishers limit the publication size by word counts. Alternatively, a detailed protocol can be published separately using open-access platforms such as https://www.protocols.io/ (accessed on 29 September 2021) or https://protocolexchange.researchsquare.com/ (accessed on 29 September 2021). Notably, no ABR protocol has yet been posted there to date.

Our work is not free of limitations. Even though 125 research groups and their corresponding authors were identified as appropriate and included in the invite, only 18 researchers participated in the survey. Such a small response can be explained because the email addresses were no longer valid in some cases. In some other, researchers initially responded positively but finally did not participate in the survey. In addition, many have likely disposed of emails offering collaborative efforts because of being floated with spam mail inviting submissions to bogus journals or participating in doubtful conferences. Therefore, a communication of an intention for collaborative work on ABR guidelines could in the future be communicated in an open-access, renowned journal, via professional social networks, such as LinkedIn or ResearchGate, and during respective conferences and meetings.

Despite the limitations, we strongly believe that our contribution could increase the quality of data reporting in auditory research. Reporting the parameters discussed should improve the reproducibility of studies. In addition, the results obtained by different research groups could be better compared and discussed. Lastly, the impact of parameters identified in our work on the ABR outcomes calls for particular attention and could initiate a distinct research trend in translational audiology.

## 5. Conclusions

The presented analysis of protocols using ABR techniques in rats revealed significant differences in the experimental approach of various research groups. The identified differences drew our attention to their importance for the final results obtained using ABR. With the help of a literature review, we have confirmed our assumptions about the following domains of significance to ABR: animal-related, equipment-related, and experimental design-related domains. Within these domains, we identified specific factors that may affect the ABR. Based on our observations, we developed recommendations for reporting the domains and their specific parameters in scientific publications. Using these recommendations in published research should increase the reproducibility of experiments using ABR on rats, contribute to a better understanding of the results obtained, and reduce the number of experimental animals used.

## Figures and Tables

**Table 1:** Usage of animal strain; gender and age of animals, as reported by research groups.

Strain									
Wistar (used in 8 studies)		Sprague-Dawley (used in 6 studies)		Long-Evans (used in 2 studies)		Fischer 344, Fischer344/NHsd (used in 2 studies)		Lewis (used in 1 study)	
outbred, albino		outbred, albino		outbred, pigmented		inbred, albino		inbred, albino	
**Gender**									
male	female	male	female	male	female	male	female	male	female
**Age (Range in Months)**									
1–6 7–18		1–3		1–3		3–24	1–1.5 and 3–24	Not used	1–1.5

**Table 2:** Reported methods for assessment of anesthesia depth.

Usage of Reflexes	Number of Reporting Protocols
eyelid reflex	7
toe reflex	13
tail-flick reflex	4
nose and vibrissae	5

**Table 3:** Anesthesia monitoring.

Anesthesia Monitoring	Number of Reporting Protocols
heart rate	2
body temperature	9
mucosal membrane color	3
breathing frequency	9

**Table 4:** Recommendation on reporting specific domains for the manuscripts concerning ABR in rats.

Domains	Factors	Recommended to Be Reported in the Central Part of the Publication (Materials and Methods)	Recommended to Be Reported as Supplementary Information
Animal-dependent domain	Animals used	strain, age, number of animals *, gender, vendor, feeding *	Exclusion criteria: observations regarding a general state of health of animals (abnormal appearance, tumors, inner ear infections, tympanic membrane perforations) *
	Animal housing	Reversed day/night cycle, number of rats per cage, acclimatization time	Conditions in the animal facility (temperature, humidity), the beginning of the light and dark phase, type and size of the cage
	Transfer of rats to the experimental area	-	Acclimatization time to the experimental area, number of rats transferred to the experimental room, a method for stress assessment
	Tympanic membrane evaluation	Performing otoscopy	-
Equipment-dependent domain	The system used to measure ABR	Company name, year of purchase	-
	Experimental area	Usage of Faraday cage	Size of the room or chamber where ABR is performed
	Experimental room temperature	The temperature in the experimental room	
	Animal’s body temperature	Body temperature during experiments; describe the monitoring system	
	Monitoring of the body temperature	The monitoring system for rat’s body temperature	
Experiment-dependent domain	Time of ABR measurement	Time and length of ABR measurement	-
	Anesthesia	Anesthetics (dosage), route of administration	Anesthesia monitoring, covering eyes
	Experimental design	Number of ABR measurement sessions per rat	The recovery process after anesthesia
	ABR analyses parameters	threshold, latency, amplitude; the number of ABR traces at near-threshold intensity	
	ABR acquisition characteristics	Signal delivery, electrodes: type, impedance, number, placement, the methods of placement (e.g., surgically fixed, subdermal)	Usage time of electrodes, disinfecting agent for electrodes
	Stimulus characteristics	Sound stimuli (type, duration, number of cycles), tested frequencies, intensity range, repetition rate, number of averageness, filters, usage of notch filters, sampling rate	

* Data not collected with a questionnaire.

## Data Availability

All data were submitted as Supplementary Materials.

## Supplementary Materials

The following are available online at https://www.mdpi.com/article/10.3390/brainsci11121596/s1, Supplementary Table S1: A questionnaire sent to the identified researchers Supplementary Table S2: Summary of the extracted data obtained from 18 participating research groups.
